# Event and Comment

**Published:** 1907-04-15

**Authors:** 


THE
### DENTAL REGISTER.

Vol. LXI. April 15, 1907. No. 4.

### EVENT AND COMMENT.

### Definite Dosage.

The following letter raises a question of importance that is worthy of especial, comment and we print it here that we may make proper and conspicuous reparation for an unintentional criticism of a very proper comment on the use of a drug of the greatest value to dentists, and which we do not all use as we should to get its greatest benefits.

"EDITOR DENTAL REGISTER: I was of the opinion that you always saw things as they should be seen, but from your discussion of Dr, Straith's paper, reported in the March REGISTER, page 138, I fear you do occasionally get things mixed. You say in your comment, that "one of the letters stated that its writer always used cocain solutions in dosage of definite parts of a grain regardless of per cent solution." Now what I really said was we do not administer per cent solutions of these drugs, but use definite dosage in parts of a grain. This did not say or mean that we gave no heed to the dilution of the solution, but that we are careful as to the amount of the drug given. It is common for dentists to say that they inject a two or four per cent solution, giving no idea as to the quantity injected. Enough of a four per cent solution would kill a horse, but one fourth of a grain properly controlled will kill no adult in good physical condition. The point I intended to make was the importance of knowing how much of the drug was given.—*Thomas L. Gilmer.*

Now here is a place where two statements were made with the best of intentions, and yet, because of a lack of clearness, or rather of completeness in their statement, they seem to have each miscarried. We most cordially agree with Dr. Gilmer's statement as he now puts it in his letter, and we have little doubt that he will agree with our comment when he adjusts it to his own experience. We had no idea of disregarding the accepted physiological dose of cocain as determined by the pharmacologist, any more than had Dr. Gilmer, what we wished to emphasize was that safer or better physiologic results should follow the use of the drug when largely diluted, taking for granted that no one would exceed the proper therapeutic dose, in drug quantity. We regret our erroneous conclusion and unfair comment on Dr. Gilmer's very proper statement, at the same time we most cordially thank him for putting this matter in its correct light. We are learning to make great use of this valuable but powerful drug and any information that experience in its use may have developed should be put before the profession in its clearest form. We believe of course in using the minimum amount of the drug, and want to make it clear that we have found from a large experience with the drug that there are few dental uses for cocain that call for more than a one per cent solution. In fact we believe that a proper dose diluted to one half per cent will answer all demands for adequate anesthesia when used for either the "high" or "low" hypodermatic pressure injections. In a few cases a more concentrated solution may be required, such as for local applications to be absorbed by the tissues by normal processes.

### Occlusion in Crown and Bridge-work.

Judging by the many failures to establish bridge work of a easonable durability as met in daily observation and as reported in the current literature, one is lead to inquire why it is not practicable to construct bridges for longer periods of usefulness than now seems possible. Among the reasons given for failures in this particular, none has been more generally advanced than that of faulty work or design. Facings break away on slight provocation, abutment teeth loosen and the whole structure becomes useless, sometimes the framework breaks or becomes distorted. There can be no question that faulty placement of material so as to withstand stress, will account for some failures, but we are coming to believe that faulty occlusion, especially in regard to the direction of force applied in the use of the teeth, has more to do with the loss of facings, and the disease of the abutment teeth than all other faults of design or construction. The reason for this we take it is all because our bridges are designed and,constructed on models made from partial and indifferently made impression. These are placed on the small hinge form of articulators, which permit of no other than the open and shut function. Can we not learn a lesson of value here from the Orthodontists, who are not satisfied with anything less than an absolutely perfect study plaster model of every case they undertake to treat, which they examine with the greatest care before undertaking any treatment. If we should secure such models and place them accurately by means of the face bow, on a good anatomical articulator and study the design for our bridge before attempting its construction, we believe our bridges would not only be made more perfectly trom the mechanical point ot view, but they would be designed to meet varying directions ot impact which tend to disturb the abutment teeth or to throw undue strain on the bridge so that it soon breaks down or must be replaced.

